# Combining the deployment of only the distal basket segment of the EMBOTRAP III and an aspiration catheter for M2 occlusions: the ONE-SEG technique

**DOI:** 10.3389/fneur.2024.1424030

**Published:** 2024-08-27

**Authors:** Yuki Hamada, Hideki Matsuoka, Shinsuke Sato, Yutaro Kawabata, Kana Iwamoto, Mei Ikeda, Takeo Sato, Go Takaguchi, Hiroshi Takashima

**Affiliations:** ^1^Department of Strokology, Stroke Center, National Hospital Organization Kagoshima Medical Center, Kagoshima, Japan; ^2^Department of Neurosurgery, St. Luke’s International Hospital, Tokyo, Japan; ^3^Department of Neuroendovascular Therapy, St. Luke’s International Hospital, Tokyo, Japan; ^4^Department of Neurology and Geriatrics, Kagoshima University Graduate School of Medical and Dental Sciences, Kagoshima, Japan

**Keywords:** thrombectomy, stroke, M2 occlusion, intervention, MeVO, DMVOs

## Abstract

**Background:**

Endovascular therapy (EVT) for distal medium vessel occlusions requires prioritizing effectiveness and safety. We developed a technique combining the deployment of only the distal basket segment of the EMBOTRAP III and an aspiration catheter (AC) for M2 occlusions, called the “ONE-SEG technique,” and evaluated its clinical and technical impacts.

**Methods:**

This was a retrospective review of 30 consecutive patients with M2 segment middle cerebral artery occlusion treated using the ONE-SEG technique. This method involves deploying the EMBOTRAP III through a microcatheter in only one segment and guiding the AC to the M2 origin or distal M1. The rates of final-pass expanded thrombolysis in cerebral infarction (eTICI) scores of 2c/3 or 2b/2c/3, safety (symptomatic intracranial hemorrhage [sICH]), and clinical outcomes (modified Rankin Scale [mRS] score 0–2, 0–3 at 90 days, and mortality at 90 days) were evaluated.

**Results:**

Of the 30 cases, 36.7% were female, and the mean age was 75.6 ± 11.0 years. The ONE-SEG technique was used for 17 cases (56.7%, median NIHSS 10 [5–15.5]) with primary M2 occlusion and 13 cases (43.3%, median NIHSS 20 [14–22.5]) with secondary M2 occlusion after proximal thrombus removal. The successful final reperfusion rate (eTICI 2b/2c/3) was 90% overall (27/30 cases). One case (3.3%) developed sICH with secondary M2 occlusion. At 3 months, mRS scores 0–2 were seen in 64.7% of patients with primary M2 occlusion (11/17 cases) and in 23.1% (3/13 cases) with secondary M2 occlusion.

**Conclusion:**

EVT using the ONE-SEG technique appears to be safe and effective for M2 occlusion.

## Introduction

The effectiveness and safety of endovascular therapy (EVT) for large vessel occlusion (LVO) in the anterior circulation have been established, and it is now considered standard treatment (1–5). However, questions still remain regarding the efficacy and safety of EVT for peripheral cerebral artery occlusions such as medium vessel occlusions (MeVOs), which account for 25–40% of all acute ischemic stroke cases (6). This is believed to be due to the challenges of medium cerebral artery occlusion treatment, since peripheral vessels are narrow, tortuous, and fragile, increasing the risk of bleeding complications associated with EVT. A meta-analysis of EVT for M2 occlusion by the HERMES collaboration reported modified thrombolysis in cerebral infarction (mTICI) 2b/3 in 58.2% and no symptomatic intracranial hemorrhage (ICH), but a mortality rate of 11.9% (7). Furthermore, a review of EVT for distal medium vessel occlusions (DMVOs) by Biljin et al. found mTICI 2b/3 in 77%, a symptomatic ICH rate of 5.7%, and a subarachnoid hemorrhage (SAH) rate of 8.3% (8), demonstrating a considerable risk of bleeding complications. Meanwhile, the TOPMOST registry suggested the potential benefit of EVT compared with best medical treatment with respect to early neurological improvement of P2 or P3 occlusions and reported a symptomatic ICH rate of 4.3% (9). Thus, for MeVOs and DMVOs, treatment prioritizing both effectiveness and safety is required.

Recently, evidence regarding the efficacy and safety of EVT for MeVOs and DMVOs has been increasing. Devices such as Tigertriever 13 (Rapid Medical, Yoqneam, Israel) (10), Mindframe Capture low profile (LP) device (Medtronic, Minneapolis, MN, USA) (11), Aperio (Acandis, Pforzheim, Germany) (12), Catch View mini (Catch View; Balt, Montmorency, France) (13), pREset LITE (phenoxGmbH, Bochum, Germany) (14), among others, have shown promising results for MeVO and DMVO treatment. This is presumed to be due to the improved performance and evolution of recent stent retrievers (SRs) and aspiration catheters (ACs), allowing devices to reach more peripheral vessels and achieve a good efficacy and safety profile.

However, there is a growing number of reports demonstrating safe and effective outcomes for MeVO or DMVO treatment by using modified existing SRs and ACs. Techniques such as the BEMP technique (15) and distal combined technique (16) are examples. In addition, results for the effectiveness and safety of semi-deployment of SRs for DMVO patients have also been reported (17). The EMBOTRAP III (CERENOVUS, Johnson & Johnson Medical Devices, Irvine, CA, USA) is an SR with a structure divided into five segments, designed to have a high thrombus capture rate and minimal resistance to the vessel. Its distal segment, called the distal basket, has a finely designed mesh at the tip, potentially capturing thrombi within it. This design, being only one segment, reduces the amount of metal deployed within the vessel and minimizes friction between the SR and the vessel wall, potentially contributing to fewer bleeding complications. A thrombectomy technique using only the distal basket segment of the EMBOTRAP III and an AC for M2, named the ONE-SEG technique, was evaluated, and the outcomes and technical parameters, such as hemorrhagic complications, SAHs, and spasm, of MCA M2 occlusion thrombectomy are reported.

## Subjects and methods

### Study design

This was a retrospective, cohort study. Data were extracted from the stroke database of our facility for patients who underwent EVT from April 2014 to March 2024. The retrospective selection criteria were as follows: (1) acute ischemic stroke (AIS) with a confirmed M2 occlusion on initial magnetic resonance imaging (MRI), or AIS with LVO treated with EVT, with distal occlusion; (2) National Institutes of Health Stroke Scale (NIHSS) score ≥ 1; (3) time from symptom onset to puncture of less than 24 h; and (4) EVT performed using the EMBOTRAP III device with the ONE-SEG technique. In addition, no definition has been established regarding whether M2 occlusion is LVO, MeVO, or DMVO. For convenience, M2 occlusion was not classified as LVO (which included the internal carotid artery or M1) in this paper, but as MeVO. This retrospective study was approved by the local ethics committee.

### Embotrap III

**Figure 1:**
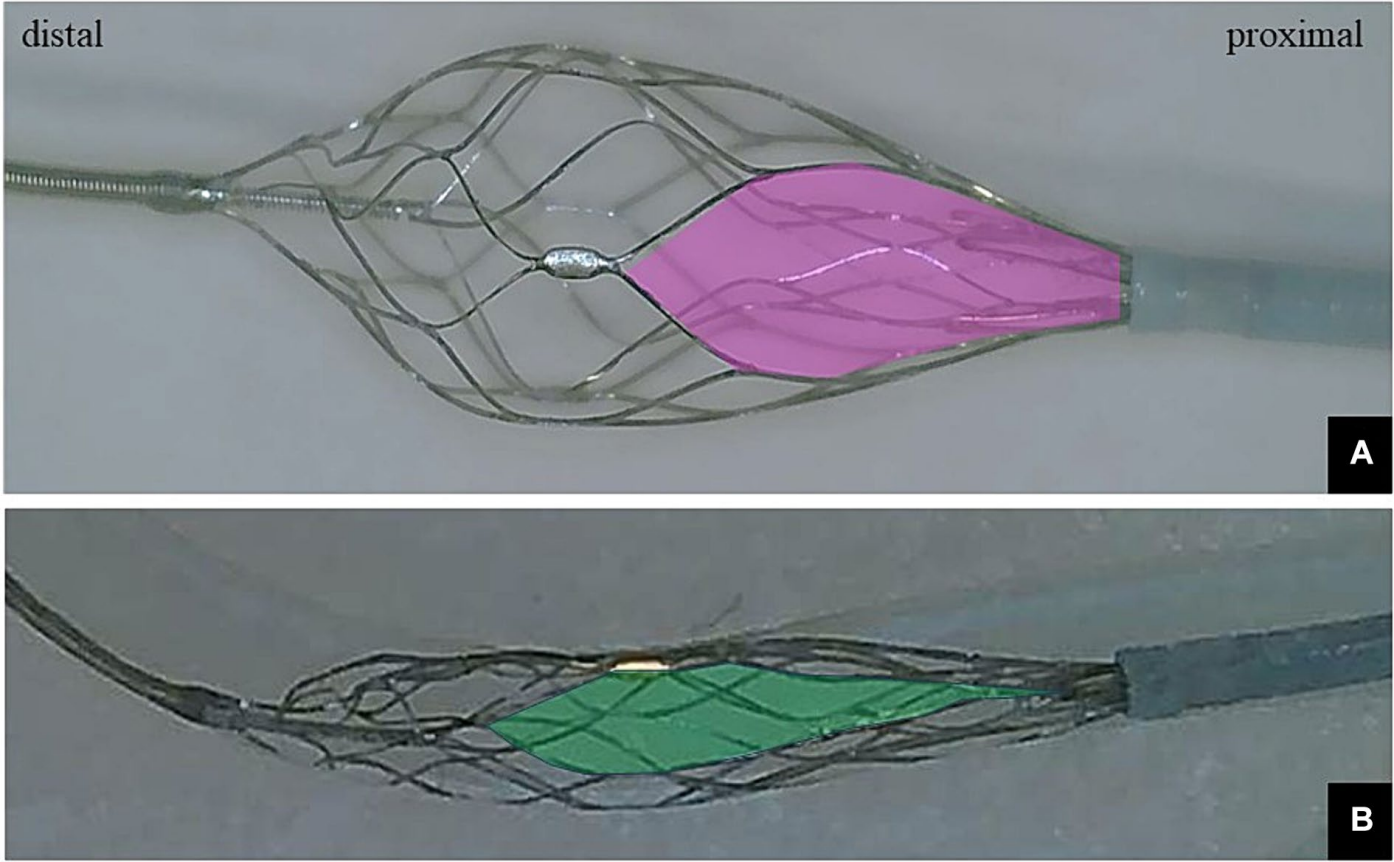
**(A)** Photograph showing only the distal basket of the EMBOTRAP III deployed. The inlet window (pink area) of the distal basket is designed to capture fragmentable thrombus. **(B)** Only the distal basket of the EMBOTRAP III is deployed in a vascular model that mimics a 2-mm-diameter M2. Compared to the inlet window in **(A)**, the inlet window (green area) in **(B)** extends in the long-axis direction.

The EMBOTRAP III reperfusion device is a dual-structure segmented SR designed to capture a wide range of thrombus compositions and adhere to the vessel wall during retrieval. The main design difference lies in the presence of closed-cell inner channels within the outer cage and distal mesh of closed cells at the tip, known as the distal basket. This design feature is expected to result in a high rate of successful revascularization in EVT (18). Whereas the distal basket is primarily designed to prevent distal embolization of thrombi, each basket is equipped with gaps called inlet windows to capture thrombi within the cage, including the distal basket, allowing for thrombus capture. An overview of the distal basket of EMBOTRAP III is shown in Figure 1.

### Interventional protocol with the ONE-SEG technique

The thrombectomy procedure was performed using the Siemens ARTIS zee biplane angiography system (Siemens, Erlangen, Germany) under local anesthesia. Cerebral digital subtraction angiography (DSA) and EVT were performed via right femoral artery puncture in all cases. A balloon-guiding catheter (BGC) was placed in the internal carotid artery on the occlusion side using either an 8-Fr Optimo (Tokai Medical Products Inc., Aichi, Japan) or an 8-Fr Emboguard (CERENOVUS, Johnson & Johnson Medical Devices).

**Figure 2:**
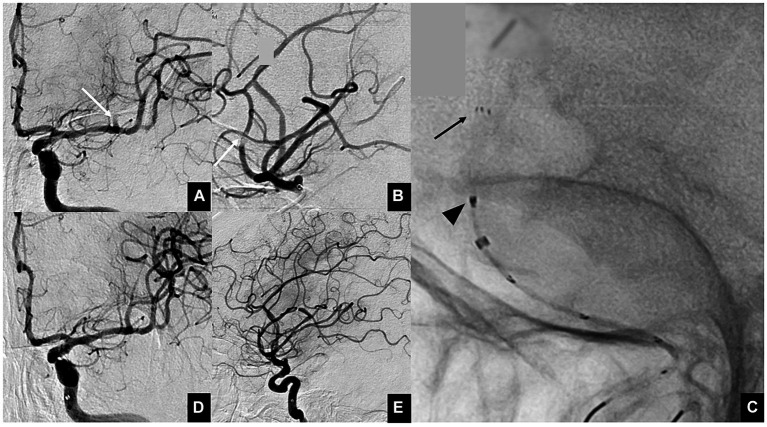
Illustrative cases of the ONE-SEG technique using an EMBOTRAP III to remove a clot. **(A)** Anterior view and **(B)** lateral view of multiple M2 occlusions. Thrombectomy is performed on the superior branch (white arrow). **(C)** Photograph showing the EMBOTRAP III deployed in a one-segment configuration (black arrow). As a guide for fluoroscopic deployment, deployment is performed up to the second marker from the tip (black arrowhead). **(D,E)** Final angiographic images, anterior view **(D)** and lateral view **(E)**, after reperfusion.

**Figure 3:**
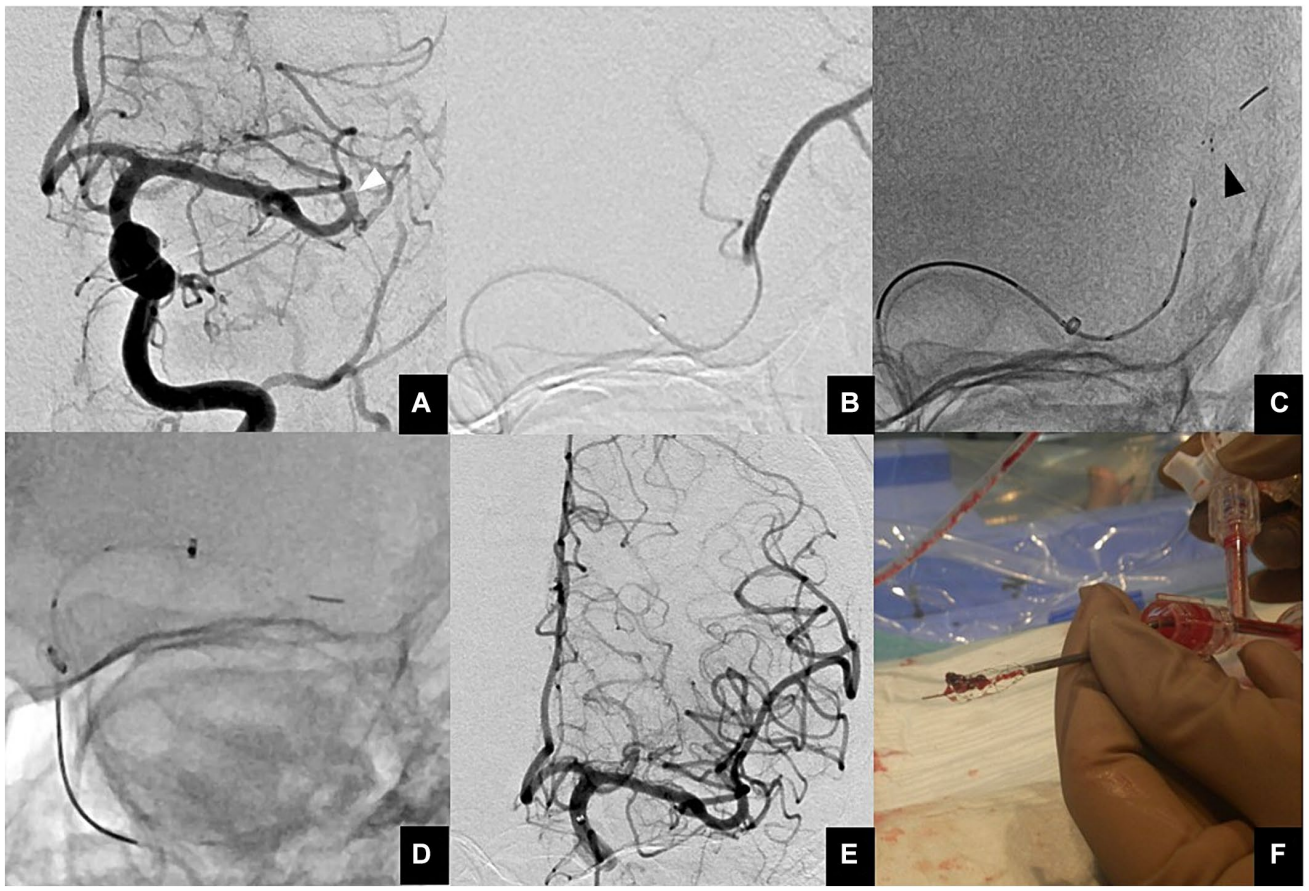
**(A)** Anterior view of a proximal M2 occlusion (white arrowhead). **(B)** A microcatheter is guided distal to a thrombus using a microguidewire. Then, contrast injection is performed to ensure that the tip of the microcatheter is beyond the thrombus. **(C)** The EMBOTRAP III is subsequently deployed in a one-segment configuration (black arrowhead). As a guide for fluoroscopic deployment, deployment is performed up to the second marker from the tip. **(D)** Since the AC cannot advance to M2, both the AC and EMBOTRAP III distal basket are pulled back to M1, and then the distal basket is pulled back into the AC. **(E)** Final angiographic image after reperfusion. **(F)** Actual thrombus retrieved within the distal basket using the ONE-SEG technique.

**Figure 4:**
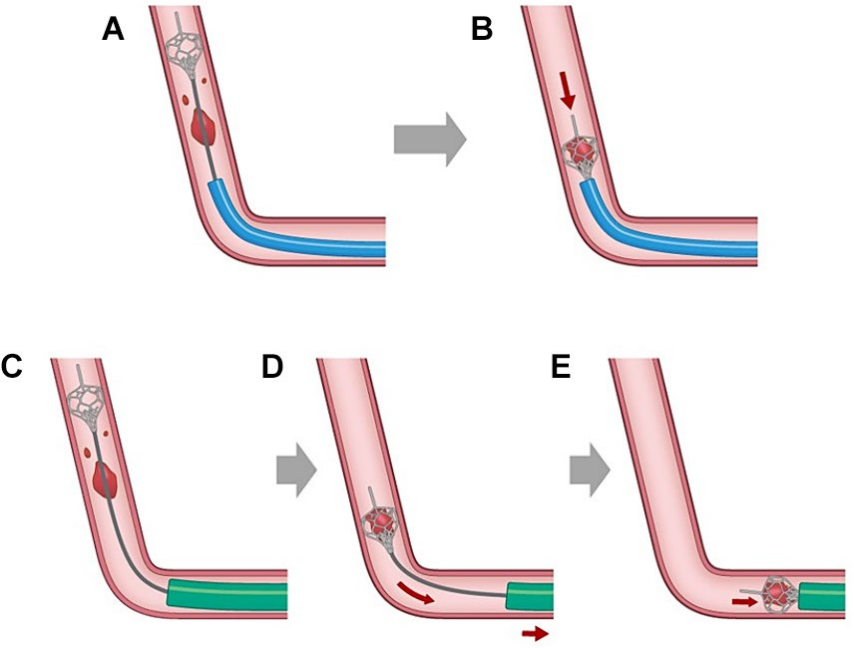
Illustration demonstrating clot retrieval using the ONE-SEG technique with the EMBOTRAP III. The balloon of the guiding catheter with a balloon is inflated to preclude flow from the internal carotid artery beforehand. **(A,B)** When the aspiration catheter (AC) can be navigated to the M2, a 0.021-inch microcatheter is guided distally using a microguidewire into the occluded vessel. Subsequently, only the distal basket of the EMBOTRAP III is deployed, with fluoroscopy guidance to ensure deployment up to the second marker from the tip. Then, aspiration is started using the AC, followed by slowly pulling and retracting the EMBOTRAP III into the AC. **(C–E)** When the AC can be navigated only to the M1 distal, similar to the approach in **(A,B)**, the distal basket of the EMBOTRAP III is deployed from the distal end of the clot. Subsequently, both the AC and the EMBOTRAP III are pulled linearly to the M1, followed by retracting the EMBOTRAP III into the AC.

The ONE-SEG technique was performed in all cases using the EMBOTRAP III device in combination with an AC. First, a 0.021-inch microcatheter, Phenom21 (Medtronic, Minneapolis, MN, USA), was placed distally to the occluded vessel. The Phenom21 was advanced as distally as possible from the occlusion site to facilitate entry of the thrombus into the inlet window of the distal basket of the EMBOTRAP III. While attempting to advance the AC as far as possible to M2, if difficulty was encountered due to vessel tortuosity or size, it was positioned at the distal M1 for standby. AC size was selected by measuring the diameter of the occluded vessel in the frontal and lateral views on baseline angiography. Subsequently, only the distal basket of the EMBOTRAP III was deployed. As a guideline for deployment under fluoroscopy, deployment was performed up to the second marker from the tip. Next, aspiration was started using a commercially available aspiration pump via the AC. If the AC could be advanced to M2, the EMBOTRAP III was slowly pulled back and withdrawn into the AC. If the AC could only be advanced to the distal M1, both the AC and the EMBOTRAP III were pulled back to M1, and then the EMBOTRAP III was withdrawn into the AC. Figures 2, 3 show an example of the ONE-SEG technique in action, and Figure 4 shows the steps of the ONE-SEG technique with illustrations.

### Clinical factors

Baseline clinical characteristics were collected for the following variables: sex, age, pre-stroke modified Rankin Scale (mRS) score, medical history (hypertension, dyslipidaemia, diabetes mellitus, current smoking, ischemic stroke, atrial fibrillation), admission systolic blood pressure, baseline NIHSS score, initial symptoms such as motor hemiparesis, sensory disturbance, and aphasia, baseline Alberta Stroke Program Early CT Score (ASPECTS) on diffusion-weighted imaging (DWI), time parameters (onset-to-door time, door-to-puncture time, puncture-to-recanalization time), treatment profile (intravenous thrombolysis), presentation type due to vascular occlusion (isolated M2 occlusion, tandem occlusion, multi-vessel M2 occlusion), M2 occlusion location (proximal, distal), M2 type (dominant, codominant, non-dominant), M2 division (superior, inferior, intermediate), M2 vessel diameter, type of AC used, and stroke etiology mechanism.

### M2 definition

The MCA M2 segment is defined as the vessel from the bifurcation/branching of the middle cerebral artery (MCA) to the circular sulcus of the insula (19, 20). The proximal M2 is defined as the horizontal M2 segment within 1 cm from the bifurcation/branching of the MCA, and the distal M2 is defined as the Sylvian M2 segment from the bifurcation/branching to the circular sulcus of the insula (7, 20, 21). M2 dominance is defined as the M2 branch having a larger diameter than other branches or when the retrograde flow disturbance due to the occluded M2 branch is greater than 50% of the same MCA territory. Occluded vessels were considered co-dominant only when the diameters of the distal and proximal branches were equal, and the associated perfusion deficit was less than 50% of the MCA territory (22). Occlusions were classified based on their clinical scenarios: isolated M2 occlusion, tandem occlusion, or multi-vessel M2 occlusion. Tandem occlusion was defined as simultaneous large vessel/proximal occlusion with M2 occlusion. Multi-vessel M2 occlusion was defined as occlusion of the M2 superior trunk and M2 inferior trunk of the MCA on the same side.

### Outcomes

Technical outcomes were defined as achieving expanded Thrombolysis in Cerebral Infarction (eTICI) scores (23) of 2c/3 and 2b/2c/3 in the target M2 segment. Safety outcomes included the presence of any parenchymal hematoma (PH) [PH type 1 or 2] based on hemorrhagic transformation criteria from the ECASS (24), any intracerebral hemorrhage (ICH), symptomatic ICH (defined as any ICH including those with an increase from the baseline NIHSS score ≥ 4 points based on ECASS II criteria), subarachnoid hemorrhage (SAH) (diffuse or focal within the territory of treated artery occlusion), extravasation (defined as extravascular leakage of contrast media), degree of spasm (defined as any degree of spasm in treated vessels), embolization to the same or different territory after thrombectomy, active perfusion deficits, and 90-day mortality. Imaging evaluation of ICH and SAH was done on day 1 with head CT. Functional outcomes were defined as good (rate of mRS scores 0–2) at discharge or at 90 days, or relatively good (mRS scores of 0–3). Patients were analyzed together and then separately based on the EVT approach: ONE-SEG technique for patients with primary M2 occlusion or ONE-SEG technique for patients with secondary M2 occlusion after proximal thrombectomy with another technique.

### Statistical methods

Statistical analysis was performed using JMP version 15 (SAS Institute, Cary, NC, USA). Continuous variables, including treatment-related time, NIHSS, DWI-ASPECTS, and mRS score, are presented as median [interquartile range (IQR)] values, and non-continuous variables are reported as proportions. Ages are shown as mean and standard deviation values.

## Results

**Figure 5:**
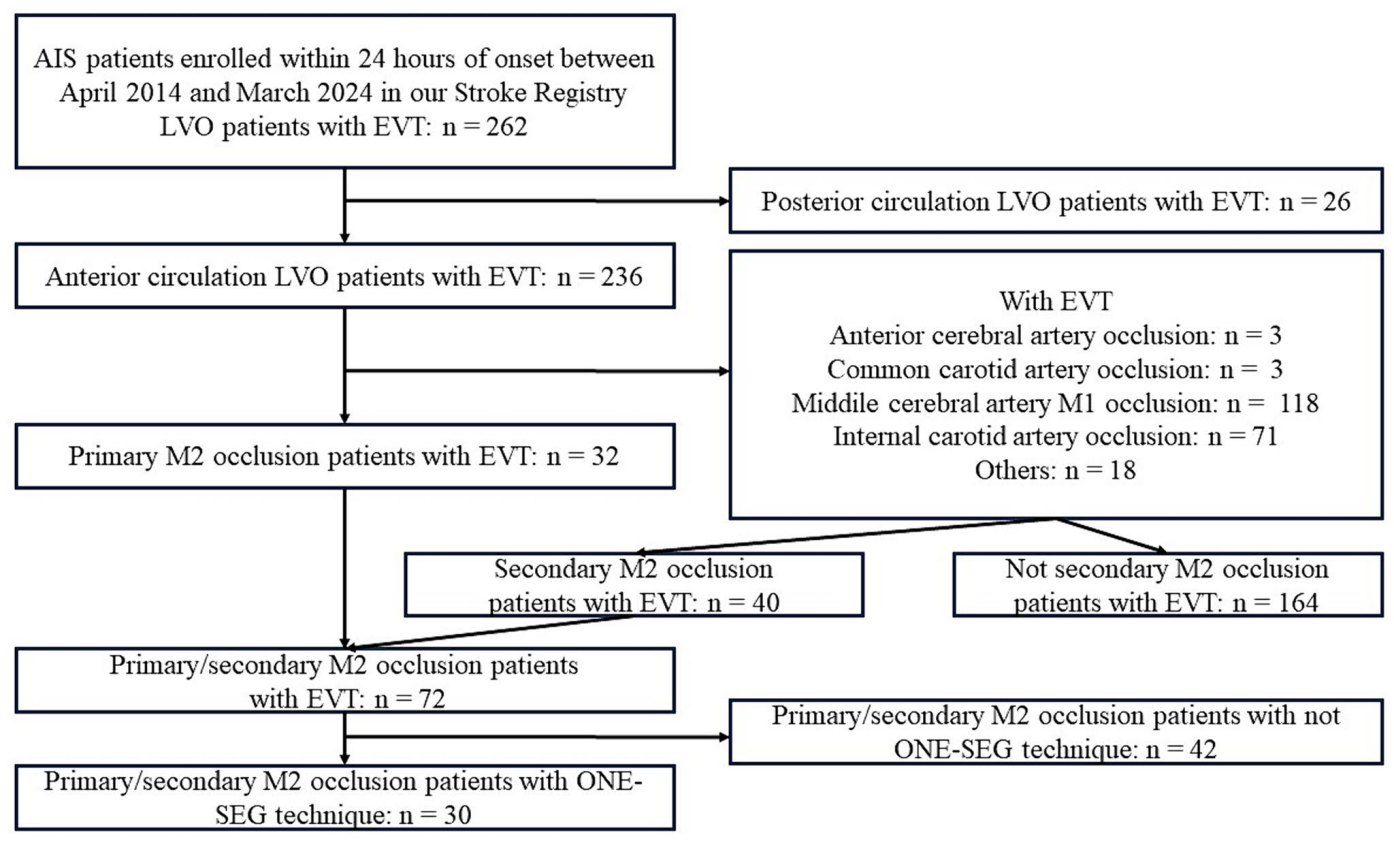
Study flowchart. AIS, acute ischemic stroke; ACA, anterior cerebral artery; CCA, common carotid artery; EVT, endovascular therapy; ICA, internal carotid artery; MCA, middle cerebral artery.

The flowchart of the study is provided in Figure 5. From a retrospective stroke registry, 262 patients diagnosed with AIS within 24 h of onset between April 2014 and March 2024 were included. Of them, 72 patients underwent EVT for M2 occlusion; 30 were treated using the ONE-SEG technique, including 17 with primary M2 occlusion and 13 with secondary M2 occlusion.

### Baseline characteristics

Results are presented in Table 1. Overall, there were 11 females (36.7%), and the average age was 75.6 ± 11.0 years. The median NIHSS score was 14 (IQR: 5.8–21.3) for all patients, 10 (5–15.5) for primary M2 occlusion, and 20 (14–22.5) for secondary M2 occlusion, indicating lower neurological severity in primary M2 occlusion cases. Motor hemiparesis was present in 26/30 (86.7%) overall, with sensory disturbance in 17/30 (56.7%). Only sensory disturbance was seen in 0%. Aphasia, which is considered to be functionally important, was seen in 18/30 (60.0%) overall. The DWI-ASPECTS was 8 (IQR: 6–9) overall, 8 (8–9.5) for primary M2 occlusion, and 6 (5–8) for secondary M2 occlusion, suggesting a more extensive infarct in secondary M2 occlusion cases. The median time from symptom onset-to-door was 107 (IQR: 65–356) minutes, median time from door-to-puncture was 68 (55–85) minutes, and median time from puncture-to-reperfusion was 59 (40–78) minutes. For primary M2 occlusion cases, the median time from puncture-to-reperfusion was 56 (34–72) minutes.

**Table 1:** Baseline characteristics of primary/secondary M2 patients treated with the ONE-SEG technique.

	All	Primary M2	Secondary M2
**Number of patients, *n* (%)**	**30 (100)**	**17 (56.7)**	**13 (43.3)**
**Baseline characteristic**			
Age, y (mean ± SD)	75.6 ± 11.0	74.9 ± 12.8	76.6 ± 8.6
Female, *n* (%)	11 (36.7)	7 (41.2)	4 (30.8)
Prestroke mRS score, median (IQR)	0 (0–0.25)	0 (0–0)	0 (0–3)
Hypertension, *n* (%)	23 (76.7)	13 (76.5)	10 (76.9)
Dyslipidaemia, *n* (%)	9 (30)	6 (35.3)	3 (23.1)
Diabetes mellitus, *n* (%)	8 (26.7)	4 (23.5)	4 (30.8)
Current smoking, *n* (%)	8 (26.7)	6 (35.3)	2 (15.4)
Ischemic stroke, *n* (%)	4 (13.3)	2 (11.8)	2 (15.4)
Atrial fibrillation, *n* (%)	22 (73.3)	12 (70.6)	10 (76.9)
Systolic blood pressure on admission, mmHg (IQR)	168 (153–178)	166 (145–184)	168 (161–178)
Baseline NIHSS score, median (IQR)	14 (5.8–21.3)	10 (5–15.5)	20 (14–22.5)
Motor hemiparesis, *n* (%)	26 (86.7)	14 (82.4)	12 (92.3)
Sensory disturbance, *n* (%)	17 (56.7)	8 (47.1)	9 (69.2)
Aphasia, *n* (%)	18 (60.0)	8 (47.1)	10 (76.9)
Baseline DWI ASPECTS score, median (IQR)	8 (6–9)	8 (8–9.5)	6 (5–8)
Intravenous alteplase, *n* (%)	13 (43.3)	10 (58.8)	3 (23.1)
**Time parameters**			
Onset-to-Door time, min (IQR)	107 (65–356)	78 (54–243)	197 (78–663)
Door-to-Puncture time, min (IQR)	68 (55–85)	71 (56–84)	62 (55–86)
Puncture-to-Recanalization time, min (IQR)	59 (40–78)	56 (34–72)	67 (45–108)

Data are presented as number (%) or median (interquartile range) values. Ages are shown as mean and standard deviation values. ASPECTS, Alberta Stroke Program Early Computed Tomographic Score; DWI, diffusion-weighted imaging NIHSS, National Institutes of Health Stroke Scale. ONE-SEG technique, combining the deployment of only the distal basket segment of the EMBOTRAP III and aspiration catheter. Bold values in Primary M2, M2 occlusion that occurred de novo; Secondary M2, M2 occlusion from occlusion of a large vessel due to migration or fragmentation of a thrombus.

Intravenous alteplase was used in 13 patients (43.3%), with a higher rate observed in primary M2 occlusion cases (10/17, 58.8%) than in secondary M2 occlusion cases (3/13, 23.1%).

The following points are presented in Table 2. BGC was used in all cases. As for the ACs, mid-sized catheters including 3MAX (Penumbra, Alameda, CA, USA) (2 cases, 6.7%), 4MAX (Penumbra) (4 cases, 13.3%), and 5Fr SOFIA (MicroVention Terumo, Aliso Viejo, CA, USA) (1 case, 3.3%) were used, along with large-sized catheters such as CAT60 (Stryker Neurovascular, Fremont, CA, USA) (1 case, 3.3%), RED62 (Penumbra) (2 cases, 6.7%), REACT68 (Medtronic, Minneapolis, MN, USA) (7 cases, 23.3%), REACT71 (Medtronic) (12 cases, 40%), and 6Fr Esperance Distal Access Catheter 0.071″ (Wallaby, Shanghai, China) (1 case, 3.3%). Of the occlusion patterns, solitary M2 occlusion accounted for 23/30 cases (76.7%). Tandem occlusion was absent, but multi-vessel M2 occlusion was observed in 7/30 cases (23.3%), although EVT was not performed for non-eloquent occlusion sites. Regarding the location of M2 occlusion, proximal and distal M2 occlusions were observed in 15/30 cases (50%) each. A dominant upper or lower branch was observed in 19/30 cases (63.3%), whereas co-dominance of M2 branches was observed in 11/30 cases (36.7%) in patients with M2 occlusion, with primary M2 in 35.3% and secondary M2 in 38.5%. Non-dominant M2 accounted for 0% throughout this study.

**Table 2:** Baseline angiographic type and stroke etiology of primary/secondary M2 patients treated with the ONE-SEG technique.

	All	Primary M2	Secondary M2
**Number of patients, *n* (%)**	**30 (100)**	**17 (56.7)**	**13 (43.3)**
**Angiographic type of presentation (by vessel)**			
Isolated M2 occlusion, *n* (%)	23 (76.7)	12 (70.6)	11 (84.6)
Tandem M2 occlusion, *n* (%)	0 (0)	0 (0)	0 (0)
Multi-vessel M2 occlusion, *n* (%)	7 (23.3)	5 (29.4)	2 (15.4)
Vessel diameter of M2, median, (IQR) mm	2.0 (1.8–2.2)	2.0 (1.9–2.3)	2.0 (1.8–2.1)
**M2 occlusion site**			
Proximal M2, *n* (%)	15 (50)	10 (58.8)	5 (38.5)
Distal M2, *n* (%)	15 (50)	7 (41.2)	8 (61.5)
**M2 division**			
Superior M2, *n* (%)	16 (53.3)	8 (47.1)	8 (61.5)
Inferior M2, *n* (%)	14 (46.7)	8 (47.1)	6 (46.2)
Intermediate M2, *n* (%)	0 (0)	0 (0)	0 (0)
**M2 type**			
Dominant M2, *n* (%)	19 (63.3)	11 (64.7)	8 (61.5)
Co-dominant M2, *n* (%)	11 (36.7)	6 (35.3)	5 (38.5)
Non-dominant M2, *n* (%)	0 (0)	0 (0)	0 (0)
**Aspiration catheter**			
3MAX, *n* (%)	2 (6.7)	1 (5.9)	1 (7.7)
4MAX, *n* (%)	4 (13.3)	2 (11.8)	2 (15.4)
5Fr SOFIA, *n* (%)	1 (3.3)	1 (5.9)	0 (0)
CAT60, *n* (%)	1 (3.3)	1 (5.9)	0 (0)
RED62, *n* (%)	2 (6.7)	2 (11.8)	0 (0)
REACT68, *n* (%)	7 (23.3)	4 (23.5)	3 (23.1)
REACT71, *n* (%)	12 (40)	5 (29.4)	7 (53.9)
6Fr Esperance, *n* (%)	1 (3.3)	1 (5.9)	0 (0)
**Etiology**			
Cardioembolic, *n* (%)	25 (83.3)	16 (94.1)	9 (69.2)
Large artery atherosclerosis, *n* (%)	4 (13.3)	3 (5.9)	3 (23.1)
Undetermined, *n* (%)	1 (3.3)	0 (0)	1 (7.7)

Data are presented as number (%) or median (interquartile range) values. Bold values in Primary M2, M2 occlusion that occurred de novo; Secondary M2, M2 occlusion from occlusion of a large vessel due to migration or fragmentation of a thrombus.

### Functional outcomes

Of the 30 patients, 6 (20%) achieved eTICI score 2c/3 on the first pass, and 12 (40%) achieved eTICI score 2b/2c/3 on the first pass. A final eTICI score of 2c/3 was achieved in 22 patients (73.3%), whereas a final eTICI score of 2b/2c/3 was obtained in 27 patients (90%). The median (IQR) number of passes for primary M2 occlusion was 2 (1–3). For secondary M2 occlusion, considering the first attempt after the occlusion changed due to the preceding technique as the 1st pass, the median (IQR) number of passes was 2 (1–2.5).

### Technical parameters

Regarding safety and efficacy outcomes, there were a total of 5 cases of ICH, with 3/5 cases (17.7%) occurring in primary M2 occlusion and 2/5 cases (15.4%) in secondary M2 occlusion cases. Symptomatic ICH occurred in 1 case (3.3%) overall, in a case of secondary M2 occlusion. PH occurred in 2 cases (6.7%) overall, all of which were observed in secondary M2 occlusion cases. Embolization to the same territory after thrombectomy for proximal occlusion was observed in 1 case (3.3%) overall, in a primary M2 occlusion case. Spasm was observed in 1/30 cases (3.3%) overall, in a primary M2 occlusion case. SAH, extravasation, and embolization to different territories were not observed.

The rate of good outcomes (mRS score 0–2) at discharge was 43.3% overall (13/30 patients), and at 3 months, it was 46.7% (14/30 patients). The rate of relatively good outcomes (mRS score 0–3) was 60% (18/30 patients) at discharge and 73.3% (22/30 patients) at 3 months. Two patients with a history of severe heart failure died. Outcomes of M2 patients treated with the ONE-SEG technique are summarized in Table 3.

**Table 3:** Outcomes of primary/secondary M2 patients treated with the ONE-SEG technique.

	All	Primary M2	Secondary M2
**Number of patients, *n* (%)**	**30 (100)**	**17 (56.7)**	**13 (43.3)**
**Outcome**			
**Procedural outcome**			
Number of passes, median (IQR)	2 (1–3)	2 (1–3)	2 (1–2.5)
First-pass eTICI score 2c/3, *n* (%)	6 (20)	3 (17.7)	3 (23.1)
First-pass eTICI score 2b/2c/3, *n* (%)	12 (40)	7 (41.2)	5 (38.5)
Final eTICI score 2c/3, *n* (%)	22 (73.3)	12 (70.6)	10 (70.9)
Final eTICI score 2b/2c/3, *n* (%)	27 (90)	15 (88.2)	12 (92.3)
**Safety outcome**			
Any ICH, *n* (%)	5 (16.7)	3 (17.7)	2 (15.4)
Symptomatic ICH, *n* (%)	1 (3.3)	0 (0)	1 (7.7)
Parenchymal hematoma, *n* (%)	2 (6.7)	0 (0)	2 (15.4)
Subarachnoid hemorrhage, *n* (%)	0 (0)	0 (0)	0 (0)
Extravasation, *n* (%)	0 (0)	0 (0)	0 (0)
Spasm, *n* (%)	1 (3.3)	1 (5.9)	0 (0)
Embolization to distal territory, *n* (%)	1 (3.3)	1 (5.9)	0 (0)
Embolization to new territory, *n* (%)	0 (0)	0 (0)	0 (0)
Mortality at 90 days, *n* (%)	2 (6.7)	1 (5.9)	1 (7.7)
**Efficacy outcome**			
mRS score at discharge, median (IQR)	3 (1–4)	2 (0.5–3)	4 (3–4.5)
mRS score 0–2 at discharge, *n* (%)	13 (43.3)	11 (64.7)	2 (15.4)
mRS score 0–3 at discharge, *n* (%)	18 (60)	14 (82.4)	4 (30.8)
mRS score at 3 months, median (IQR)	3 (1–4)	1 (0–3)	3 (2.5–4.5)
mRS score 0–2 at 3 months, *n* (%)	14 (46.7)	11 (64.7)	3 (23.1)
mRS score 0–3 at 3 months, *n* (%)	22 (73.3)	15 (88.2)	7 (53.9)

Data are presented as number (%) or median (interquartile range) values.

eTICI, expanded Thrombolysis in Cerebral Infarction; ICH, intracranial hemorrhage; mRS, modified Rankin Scale; ONE-SEG technique, combining the deployment of only the distal basket segment of the EMBOTRAP III and aspiration catheter. Bold values in Primary M2, M2 occlusion that occurred de novo; Secondary M2, M2 occlusion from occlusion of a large vessel due to migration or fragmentation of a thrombus.

## Discussion

### Clinical outcomes and technical parameters

In this retrospective study, the ONE-SEG technique for M2 occlusion appeared to be safe and effective, both for primary and secondary M2 occlusions. In this selected small series of patients, the clinical outcomes seemed encouraging, with functional independence at 3 months observed in 13 of 30 patients overall (46.7%), with 11 of 17 patients (64.7%) with primary M2 occlusion achieving functional independence at 3 months. In contrast, only 3 of 13 patients (23.1%) with secondary M2 occlusion achieved functional independence at 3 months, but the mRS score 0–3 rate was 53.9% (7/13). This discrepancy may be attributed to the fact that patients with secondary M2 occlusion had higher baseline NIHSS scores (median 20 vs. 10), lower baseline DWI-ASPECTS values (median 6 vs. 8), and delayed onset-to-door times (median 197 vs. 78 min), which were believed to have affected the outcomes. There were only 2 deaths (6.7%), which is similar to previous reports and may suggest the clinical benefits and safety of the ONE-SEG technique for M2 occlusion. Symptomatic ICH was observed in 1 case (3.3%), and PH was observed in 2 cases (6.7%), all in cases of secondary M2 occlusion. SAH, extravasation, and spasm were not observed. Kurre et al. (14) described their experience using the pREset LITE device in patients with extensive occlusions, achieving a successful reperfusion rate (modified TICI 2b/3) of 70%, with a low rate of vasospasm (5.6%), similar to the low incidence of spasm observed in the present study (3.3%). Kühn et al. (25) used the 3 mm × 20 mm Trevo XP ProVue (Baby Trevo, Stryker Neurovascular, Fremont, CA, USA), achieving successful reperfusion in 85.7% (TICI grade 2b/3) of cases without observed vascular damage, rupture, or significant vasospasm. These results are believed to minimize stress on the vessel wall and reinforce the clinical safety of the ONE-SEG technique.

### Radiological outcomes

Of all M2 patients treated with the ONE-SEG technique, effective reperfusion with final eTICI 2b/3 was achieved in 27 of 30 cases (90%). Comparing baseline characteristics and angiographic and clinical outcomes of M2 occlusion patients treated with the ONE-SEG technique with other series focusing on DMVO, they appeared similar. Guenego et al. (10) used the Tigertriever 13 in DMVO patients, reporting an overall effective reperfusion rate (modified TICI 2b/3) of 94% (91% for primary DMVO, 100% for secondary DMVO) and good clinical outcomes (mRS score 0–2 at 3 months) of 65% (55% for primary DMVO, 83% for secondary DMVO). In addition, Dobrocky et al. (11) used the Mindframe Capture LP device for M2 occlusions, achieving a successful reperfusion rate (modified TICI 2b/3) of 74% and good clinical outcomes (mRS score 0–2 at 3 months) in 65% of cases. Hofmeister et al. (13) used a different device, the CatchMini, resulting in a successful reperfusion (good capillary reperfusion) rate of 78% and good clinical outcomes (mRS score ≤ 2) in 82.4% of cases.

### Procedural concepts

The ONE-SEG technique relies primarily on a combined technique using both an SR and an AC. Though various effective techniques have been reported as combined techniques for LVO (26–28), reports specifically focusing on techniques limited to DMVO are scarce (15, 16). The ONE-SEG technique is divided into two methods based on whether the AC can be advanced to the M2 segment or only to the distal M1 segment. This is to align the vector of traction of the SR with the aspiration port of the AC. If the AC can only be advanced to the distal M1 segment, there will be a misalignment of the aforementioned axis, potentially leading to clot loss when retracting the SR into the AC. Therefore, both devices are initially lowered to the M1 segment of the MCA, which is anatomically closer to a straight line, before retracting the SR into the AC. This method is believed to prevent a decrease in thrombus retrieval efficiency.

In addition, the structure of the EMBOTRAP III is unique compared to other SRs. The distal basket of the EMBOTRAP III has an inlet window, a gap designed into the distal basket. Compared to the previous EMBOTRAP II, the mesh at the tip of the distal basket has become finer, and the gap of the inlet window has been slightly expanded in the EMBOTRAP III. Whereas the diameter of the M2 vessel is reported to be 1.4–2.3 mm (29), when deploying the device in narrow vessels for a one-segment expansion, the gap of the inlet window expands further. This structure facilitates the capture of thrombi even in small-diameter vessels and contributes to preventing distal embolization of thrombi. It is believed that this feature contributes to the successful incorporation of clot retrieval during one-segment expansion. This may explain the relatively high rate of final eTICI 2b/2c/3 observed in the present study. Furthermore, one-segment expansion reduces friction of the SR, leading to decreased bending, stretching, and twisting of small tortuous distal vessels, which is expected to prevent damage during withdrawal of penetrating branches.

### Strengths and pitfalls of the ONE-SEG technique

One of the strengths of the ONE-SEG technique is its ability to perform reperfusion procedures safely and effectively even in relatively tortuous vessels branching from the superior trunk or similar small bends, with minimal friction and resistance to the vessel during traction. Since only one of the five segments of the EMBOTRAP III is deployed, the metal contact area with the vessel is minimized, reducing the traction force on the vessel during SR retrieval and contributing to a decreased risk of penetrating branch withdrawal injury or vessel dissection. Another strength is the simplicity of the technique compared with other combined techniques, which is believed to have minimal impact on the delay in puncture-to-reperfusion time. Furthermore, the EMBOTRAP III has a 4-mm flexible distal marker at the tip, which allows for advancement in tortuous vessels with minimal risk of vessel damage and enables deployment while pushing out during one-segment expansion. The authors have not seen vessel damage during push-out expansion in tortuous segments, indicating an advantage in that even non-experts can perform this technique safely.

However, there are several pitfalls to this technique. When there is a large amount of thrombus, there may be limitations to the occupied volume of the distal basket, and it may not be possible to retrieve all thrombi in one pass. Therefore, it is recommended that the procedure be performed in multiple passes rather than insisting on reperfusion in one pass, especially when there is a large amount of thrombus. The median number of passes in ONE-SEG technique was two. In addition, it has been reported that it may be difficult to achieve successful reperfusion in cases of firm clot (30). It should also be noted that, depending on the location of the thrombus (e.g., on the convex or concave side of the vessel), there may be difficulty in effectively capturing it with the inlet window of the distal basket. Thus, achieving reliable retrieval may also be challenging with this technique.

### Limitations

There are several limitations to this study. First, it was a single-arm study with a small sample size of 30 cases. Second, the ONE-SEG technique using the EMBOTRAP III cannot be compared to techniques using other devices, including simple SR techniques, AC, or the combined use of stent retriever techniques. Third, the lack of a formal protocol for device selection may introduce bias by not measuring variables not captured for the ONE-SEG technique for distal occlusion thrombectomy. Fourth, the inclusion criteria of NIHSS score ≥ 1 point and within 24 h of onset may overestimate the efficacy of EVT for M2 occlusions. Fifth, the present study included M2 occlusion cases with an NIHSS score ≥ 1. However, the efficacy of EVT for mild symptoms with an NIHSS score ≤ 5 has not been demonstrated. For MeVO cases, though intravenous rt-PA therapy may be effective, the comparative benefit of monotherapy versus combination therapy with EVT in cases with mild symptoms remains unclear. Sixth, although this procedure is based on the premise of a combined technique, it is considered to be safe and easy to perform even for first-time students of thromboprophylaxis because of its low intraoperative complications. However, its widespread adoption depends on medical infrastructure, expertise, and patient choice. Continued research and education are essential to increase its accessibility and impact. Finally, due to insurance issues, careful consideration of the costs associated with device use is necessary. However, despite these limitations, the present results are encouraging, and further research is warranted.

## Conclusion

Using the ONE-SEG technique for thrombus extraction appears to be safe and effective for M2 occlusions, classified as MeVOs. Clinical outcomes, including high rates of successful reperfusion and minimal hemorrhagic complications, align with those observed in patients with proximal occlusions. However, larger cohort studies are needed to verify its clinical benefits.

## Data Availability

The original contributions presented in the study are included in the article/supplementary material, further inquiries can be directed to the corresponding author.
